# Comparison of the out-of-pocket costs of Medicare-funded telepsychiatry and face-to-face consultations: A descriptive study

**DOI:** 10.1177/10398562241237128

**Published:** 2024-03-04

**Authors:** Luke S-C Woon, Stephen Allison, Tarun Bastiampillai, Steve Kisely, Paul Maguire, William Pring, Rebecca Reay, Jeffrey CL Looi

**Affiliations:** Academic Unit of Psychiatry and Addiction Medicine, School of Medicine and Psychology, Canberra Hospital, 102945 The Australian National University Medical, Canberra, ACT, Australia; and; Department of Psychiatry, Faculty of Medicine, The National University of Malaysia, Kuala Lumpur, Malaysia; Consortium of Australian-Academic Psychiatrists for Independent Policy and Research Analysis (CAPIPRA), Canberra, ACT, Australia; and; College of Medicine and Public Health, 1065 Flinders University, Adelaide, SA, Australia; Consortium of Australian-Academic Psychiatrists for Independent Policy and Research Analysis (CAPIPRA), Canberra, ACT, Australia;; College of Medicine and Public Health, 1065 Flinders University, Adelaide, SA, Australia; and; Department of Psychiatry, Monash University, Clayton, VIC, Australia; Consortium of Australian-Academic Psychiatrists for Independent Policy and Research Analysis (CAPIPRA), Canberra, ACT, Australia;; School of Medicine, Princess Alexandra Hospital, 1974 University of Queensland, Brisbane, QLD, Australia; and; Departments of Psychiatry, Community Health and Epidemiology, Dalhouise University, Halifax, NS, Canada; Academic Unit of Psychiatry and Addiction Medicine, School of Medicine and Psychology, Canberra Hospital, 102945 The Australian National University Medical, Canberra, ACT, Australia; and; Consortium of Australian-Academic Psychiatrists for Independent Policy and Research Analysis (CAPIPRA), Canberra, ACT, Australia; Consortium of Australian-Academic Psychiatrists for Independent Policy and Research Analysis (CAPIPRA), Canberra, ACT, Australia; Department of Psychiatry, Monash University, Melbourne, VIC, Australia;; Centre for Mental Health Education and Research at Delmont Private Hospital, Melbourne, VIC, Australia; and; Egmont Terrace Specialist Rooms, Private Psychiatrist, Melbourne, VIC, Australia; Academic Unit of Psychiatry and Addiction Medicine, School of Medicine and Psychology, Canberra Hospital, 102945 The Australian National University Medical, Canberra, ACT, Australia; Academic Unit of Psychiatry and Addiction Medicine, School of Medicine and Psychology, Canberra Hospital, 102945 The Australian National University Medical, Canberra, ACT, Australia; and; Consortium of Australian-Academic Psychiatrists for Independent Policy and Research Analysis (CAPIPRA), Canberra, ACT, Australia

**Keywords:** cost comparison, out-of-pocket costs, telephone, telepsychiatry, videoconferencing

## Abstract

**Objective:**

Telepsychiatry items in the Australian Medicare Benefits Schedule (MBS) were expanded following the COVID-19 pandemic. However, their out-of-pocket costs have not been examined. We describe and compare patient out-of-pocket payments for face-to-face and telepsychiatry (videoconferencing and telephone) MBS items for outpatient psychiatric services to understand the differential out-of-pocket cost burden for patients across these modalities.

**Methods:**

out-of-pocket cost information was obtained from the Medical Costs Finder website, which extracted data from Services Australia’s Medicare claims data in 2021–2022. Cost information for corresponding face-to-face, video, and telephone MBS items for outpatient psychiatric services was compared, including (1) Median specialist fees; (2) Median out-of-pocket payments; (3) Medicare reimbursement amounts; and (4) Proportions of patients subject to out-of-pocket fees.

**Results:**

Medicare reimbursements are identical for all comparable face-to-face and telepsychiatry items. Specialist fees for comparable items varied across face-to-face to telehealth options, with resulting differences in out-of-pocket costs. For video items, higher proportions of patients were not bulk-billed, with greater out-of-pocket costs than face-to-face items. However, the opposite was true for telephone items compared with face-to-face items.

**Conclusions:**

Initial cost analyses of MBS telepsychiatry items indicate that telephone consultations incur the lowest out-of-pocket costs, followed by face-to-face and video consultations.

Medicare-funded telepsychiatry has become more widely available in Australia since the onset of the COVID-19 pandemic. The subsequent increased uptake of telepsychiatry suggests that the policy change improved access to psychiatric services.^1^ Nonetheless, out-of-pocket (OOP) costs for telepsychiatry might still create financial barriers.^2^ While patients receive reimbursement from Medicare for private psychiatric consultations, this reimbursement does not usually cover the entire consultation fee, thus incurring OOP costs. In Australia, approximately 14.7% of all healthcare costs in 2019/20 were borne by individuals (OOP expenses) compared with the following healthcare cost allocations: 42.7% by federal governments, 27.7% by state/territory governments, 8.2% by private insurance, and 6.7% by non-government organisations.^3^

For Medicare-reimbursed medical or allied health consultations, a linear relationship exists between the average OOP costs and the proportion of patients who decide not to obtain care. Higher average OOP costs for psychiatrists result in a greater proportion of patients opting out of consultations, compared with psychologists and general practitioners.^4^ While some patients will have access to bulk billing, where the psychiatrist accepts the Medicare Benefits Schedule (MBS) reimbursement as the full payment, rates of bulk billing varied greatly by state/territory and by item number.^5^ Accordingly, patients potentially lacking the financial means to access private practice telepsychiatry due to OOP costs remains a substantive concern.^6^

To date, there have been no peer-reviewed cost analyses of MBS telepsychiatry consultation items, and specifically, examination of consultation OOP costs. We describe and compare patient OOP payments for face-to-face and telepsychiatry (videoconferencing and telephone) MBS items for outpatient psychiatric services to understand the OOP cost burden for patients across different modalities of outpatient psychiatric services.

## Methods

We conducted a descriptive analysis of the OOP cost information of MBS items for outpatient psychiatric services. The OOP costs for Medicare-reimbursed private practice telepsychiatry and face-to-face consultations were derived from the Australian Department of Health and Aged Care’s Medical Costs Finder (MCF) website (https://medicalcostsfinder.health.gov.au/). The MCF shows typical private health sector costs for common services delivered out-of-hospital and in-hospital. For out-of-hospital data, the MCF uses the most recently available Medicare claims data collected by Services Australia in the financial year 2021–22 to determine the OOP costs. This is based on the invoices submitted by patients for reimbursement of private medical consultations that specify the total consultation fee charged to the patient, along with the MBS item recorded in the process.

The following information was obtained from the MCF for the relevant MBS items at the national level: (1) Median specialist fee in 2021–22; (2) Median OOP fee in 2021–22; (3) Medicare reimbursement for the service rounded to the nearest US dollar; and (4) Proportion of patients subject to OOP fees in 2021–22. The website was accessed between 13 June 2023 and 4 August 2023.

The MCF did not provide information on costs and fees for all MBS items for outpatient psychiatric consultations. To ensure proper comparisons, this study therefore only included MBS items of services that had cost information across all three categories: face-to-face, video, and telephone. The telepsychiatry (video and telephone) and face-to-face MBS items listed in Table 1 were included for analysis.

**Table 1.** Telepsychiatry and face-to-face MBS items included for OOP cost comparison

Service	MBS item		
	Face-to-face	Video	Telephone
Consultant psychiatrist. Consultation, not more than 15 min	300	91827	91837
Consultant psychiatrist. Consultation, 15 to 30 min	302	91828	91838
Consultant psychiatrist. Consultation, 30 to 45 min	304	91829	91839
Consultant psychiatrist. Consultation, 45 to 75 min	306	91830	91840^a^
Consultant psychiatrist. Consultation, more than 75 min	308	91831	91841^a^
Consultant psychiatrist, prepare a management plan, more than 45 min	291	92435	92475^a^
Consultant psychiatrist, attendance, new patient (or has not received attendance in preceding 24 months), more than 45 min	296	92437	92477^a^

^a^These telephone items were discontinued from 1 Jul 2022 onward.

To compare the proportion of patients making OOP payments versus being bulk-billed between modes of consultation, usage data of the included MBS items for the same period was downloaded from Services Australia (https://medicarestatistics.humanservices.gov.au/statistics/mbs_item.jsp). Numbers of services incurring OOP payment for all included items were derived from the services counts and OOP rates. The counts for items in each consultation mode were subsequently combined and used to calculate the overall OOP payment rates.

While information on fees and costs by state and territory was also available, it was incomplete for most of the included MBS items. This was because some regions did not show data due to insufficient services or providers available to ensure the deidentification of individual service users. Therefore, analyses at the state/territory level were not conducted. Analyses were performed using Microsoft Excel.

## Results

Table 2 summarises the OOP costs for MBS face-to-face and telepsychiatry items for 2021–2022. Medicare reimbursement amounts were the same for all comparable face-to-face and telepsychiatry items of the same duration with identical maximum and minimum Medicare reimbursements across the categories. By contrast, median specialist fees and resulting OOP payments varied across categories, these being highest for video consultation and lowest for those by telephone (Table 2). As a consequence, the proportions of patients making OOP payments ranged from 13% to 85% (For detailed results, see Supplemental Table 1).

**Table 2.** Summary of cost and fee information of MBS items for outpatient psychiatric services grouped according to modes of consultation

	Median Medicare reimbursement (Australian dollar)		Median specialist fee (Australian dollar)		Median OOP payment (Australian dollar)		Proportion of patients paying OOP (%)	
	Min	Max	Min	Max	Min	Max	Min	Max
Face-to-face	39	406	110	555	71	206	37	85
Video	39	406	115	635	76	229	40	79
Telephone	39	406	100	526	61	196	13	53

### Face-to-face items versus video items

**Figure 1.**
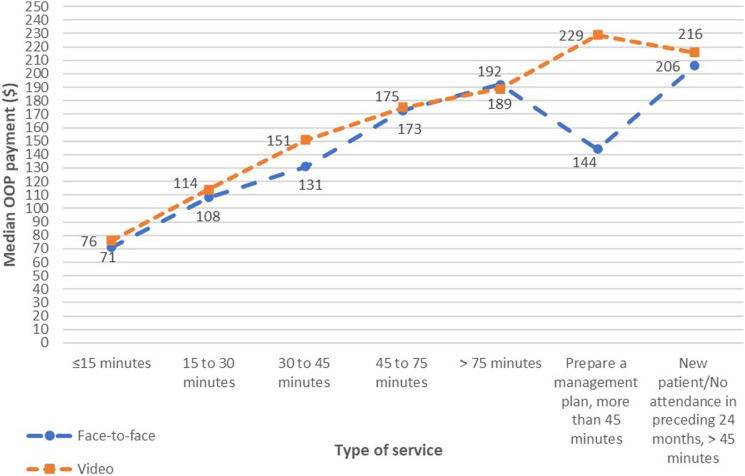
Comparisons of median OOP payments between face-to-face and video items for outpatient psychiatric services.

Comparing MBS video items with face-to-face items, median patient OOP payments for video items were consistently slightly higher than comparable face-to-face items except for consultations of more than 75 min (Figure 1).

**Figure 2.**
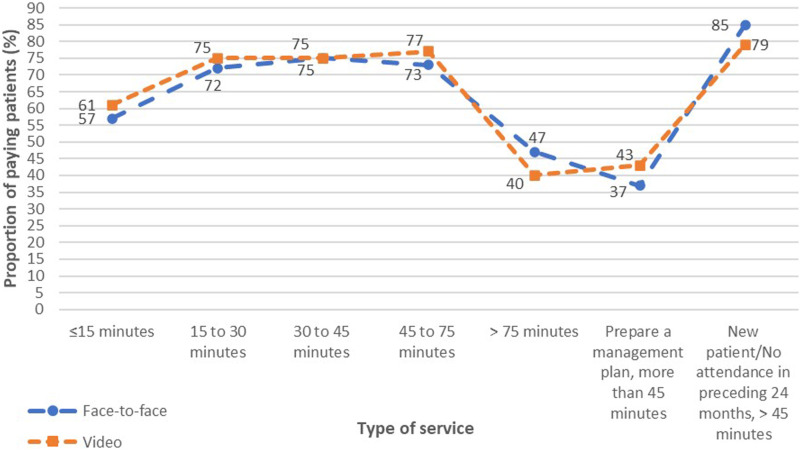
Comparisons of proportions of patients making OOP payments between face-to-face and video items for outpatient psychiatric services.

The proportions of patients charged OOP payments for video items were slightly higher than face-to-face items except for consultations of more than 75 min and new patient consultations of more than 45 min (Figure 2).

### Face-to-face items versus telephone items

**Figure 3.**
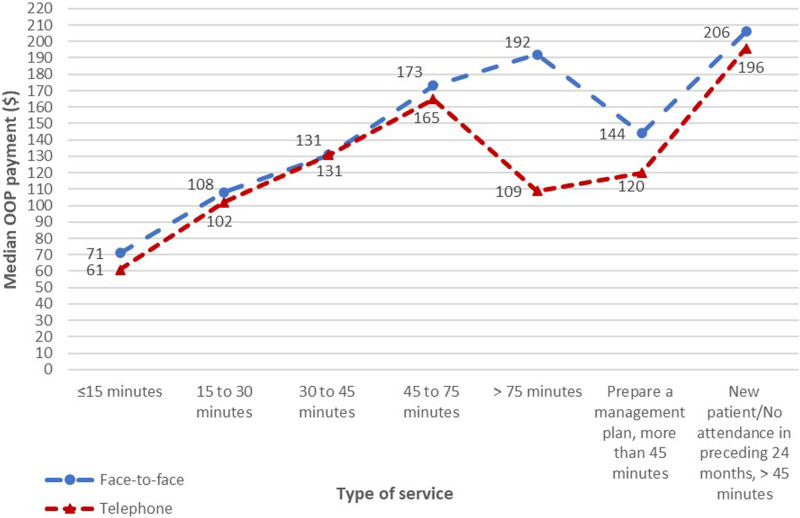
Comparisons of median OOP payments between face-to-face and telephone items for outpatient psychiatric services.

Conversely, patient OOP payments for telephone items were consistently slightly lower than comparable face-to-face items (Figure 3).

**Figure 4.**
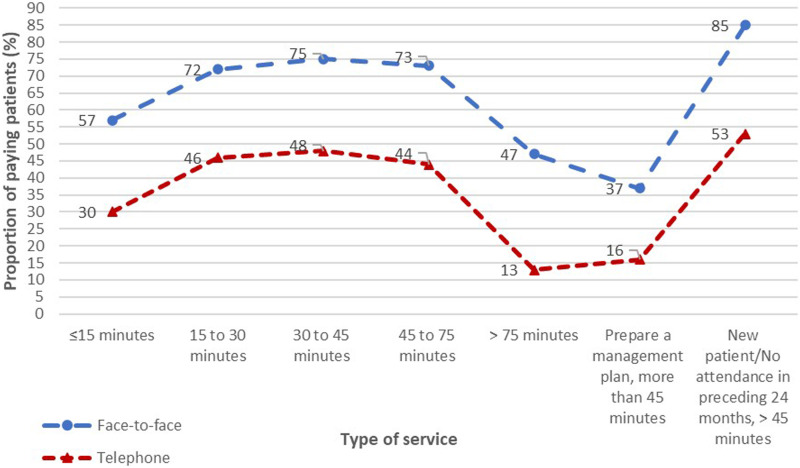
Comparisons of proportions of patients making OOP payments between face-to-face and telephone items for outpatient psychiatric services.

Additionally, the proportions of patients making OOP payments for telephone items were also consistently much lower than face-to-face items across all types of services (Figure 4).

### Comparisons of overall differences in OOP payment rates

**Figure 5.**
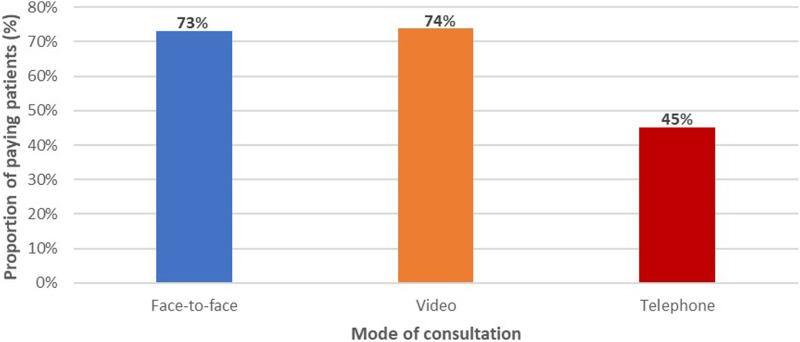
Proportions of patients making OOP payments for different modes of consultation.

As shown in Figure 5, the OOP payment rates were similar for the face-to-face (73%) and video (74%) categories, but the rate was considerably lower for the telephone category at 45% (Supplemental Table 2).

## Discussion

We examined the costs of different modes (face-to-face, video, and telephone) of MBS items for outpatient psychiatric consultations for 2021–2022. As a baseline, the MBS provided the same reimbursement to patients for the same length of consultation, whether these occurred by video, telephone or face-to-face. However, the private specialist fees for comparable consultation items varied, resulting in differences in OOP costs across these consultation modalities. Patients paying private consultation fees (those not bulk-billed) incurred slightly higher OOP costs for video items compared with face-to-face items, but telephone items incurred lower costs than either video or face-to-face consultations. The largest difference noted between these modes of service delivery was the much lower proportion of patients charged OOP expenses for telephone consultations compared with both face-to-face and video consultations.

Telepsychiatry, by videoconferencing or telephone, is attractive to many patients as it offers convenience through potentially reduced travel costs and waiting time opportunity costs in comparison with face-to-face consultation.^7,8^ However, differences in OOP costs may also affect patient preference, since higher costs may create financial barriers for access to mental health care.^9^ While the MBS video items might still be cost-saving for some patients and after additional opportunity costs are considered, telephone consultations are likely to be the cheaper options for most patients, on the basis of the lesser OOP costs for this mode of psychiatric consultation.

When these telephone telehealth item numbers came into existence during the pandemic, psychiatrists might have charged bulk-billing fees for short, telephone clinical consultations, not previously billed. This might have reduced OOP for telephone consultations overall. Rising autism and ADHD diagnoses may be related to the availability of National Disability Insurance Scheme (NDIS) support.^10^ As those with autism and ADHD may be more often seen via video consultations, this may have increased OOP costs overall.

Studies have shown that telephone and video telepsychiatry are effective and non-inferior to in-person care.^11^ In contrast, the Australian federal government rescinded reimbursement for MBS items for telephone consultations lasting 45 min or longer, from 1 July 2022.^12^ In this context, video consultations may have advantages over telephone consultations from a clinical viewpoint and may better meet the needs of certain patients.^13^ However, given the greater accessibility, and lower costs, of telephone consultations, a flexible approach that enables the utilisation of both video and telephone alternatives to face-to-face consultations would confer advantages by providing optimum patient care. This contrasts with recommendation 9 of the draft Medicare Review Advisory Committee Post Implementation of Telehealth report that promotes the abolition of patient reimbursement for all initial non-GP specialist telehealth consultations.^14^ In our view, based on the existing usage data, which at least partially reflect patient demand and preference,^6^ and with reduced OOP costs for telephone telepsychiatry, which may increase accessibility of care, this governmental abolition of reimbursement for all initial non-GP specialist consultations is regrettable as it is likely to reduce access to care, especially for rural and remote patients.

The current study has several limitations. Only comparable MBS items with available cost information across face-to-face, video, and telephone categories were included, resulting in the omission of some items from the analyses. Aggregated cost data were publicly accessible only at the national level, precluding a more detailed analysis of potential factors. For instance, cost data according to geographical areas would likely require payment to Services Australia for data access, subject to approval. Individual-level data, such as clinical complexity and socioeconomic status, were unavailable. Additional costs that may influence treatment choices, such as travel costs associated with face-to-face consultations,^15^ also could not be included. Practitioner business costs associated with telehealth were also an unknown variable.

Differences in the extent and magnitude of OOP costs between telepsychiatry and face-to-face consultations, as well as the differences between video and telephone consultations, warrant further qualitative and cost analyses, using health economic methods. The determinants of telehealth billing at the individual practitioner and patient levels are currently unknown. Analysis of OOP cost scenarios, as patients obtain either telepsychiatry or face-to-face services, may assist policymakers in the formulation of funding policy, for instance, by considering the possibility of differentiation of OOP payments between types of services,^2^ or adjusting the types of services included in the current funding scheme.

## Supplemental Material

Supplemental Material - Comparison of the out-of-pocket costs of Medicare-funded telepsychiatry and face-to-face consultations: A descriptive study Supplemental Material for Comparison of the out-of-pocket costs of Medicare-funded telepsychiatry and face-to-face consultations: A descriptive study by Luke S-C Woon, Stephen Allison, Tarun Bastiampillai, Steve Kisely, Paul Maguire, William Pring, Rebecca Reay and Jeffrey CL Looi in Australasian Psychiatry
