# Supplementary material for: Comparison of the out-of-pocket costs of Medicare-funded telepsychiatry and face-to-face consultations: A descriptive study
Source: Australas Psychiatry. 2024 Mar 4;32(3):204–9. doi: 10.1177/10398562241237128 (PMC11103898; doi:10.1177/10398562241237128)
Supplement: Supplemental Material - Comparison of the out-of-pocket costs of Medicare-funded telepsychiatry and face-to-face consultations: A descriptive study [file sj-pdf-1-apy-10.1177_10398562241237128.pdf]

**Comparison of the out-of-pocket costs of Medicare-funded telepsychiatry and face-to-face consultations: a descriptive study**

*Supplementary Table 1 Detailed information of included MBS items.*

| Service | Consultation mode | Item number | Specialist fees ($) | Medicare paid ($) | Patient paid ($) | 10th Percentile ($) | 90th Percentile ($) | Proportion of OOP payment (%) | Service count (N) | Count of services with OOP payment |
|---|---|---|---|---|---|---|---|---|---|---|
| Consultant psychiatrist. Consultation, not more than 15 minutes | Face-to-face | 300 | 110 | 39 | 71 | 36 | 96 | 57 | 17,913 | 10,210 |
| | Video | 91827 | 115 | 39 | 76 | 45 | 211 | 61 | 7,288 | 4,446 |
| | Telephone | 91837 | 100 | 39 | 61 | 31 | 96 | 30 | 33,777 | 10,133 |
| Consultant psychiatrist. Consultation, 15 to 30 minutes | Face-to-face | 302 | 190 | 78 | 108 | 52 | 152 | 72 | 141,604 | 101,955 |
| | Video | 91828 | 192 | 78 | 114 | 50 | 172 | 75 | 47,148 | 35,361 |
| | Telephone | 91838 | 180 | 78 | 102 | 42 | 147 | 46 | 116,320 | 53,507 |
| Consultant psychiatrist. Consultation, 30 to 45 minutes | Face-to-face | 304 | 250 | 120 | 131 | 56 | 181 | 75 | 415,283 | 311,462 |
| | Video | 91829 | 270 | 120 | 151 | 81 | 201 | 75 | 134,283 | 100,712 |
| | Telephone | 91839 | 250 | 120 | 131 | 61 | 176 | 48 | 187,136 | 89,825 |
| Consultant psychiatrist. Consultation, 45 to 75 minutes | Face-to-face | 306 | 340 | 165 | 173 | 54 | 260 | 73 | 305,049 | 222,686 |
| | Video | 91830 | 340 | 165 | 175 | 70 | 265 | 77 | 193,001 | 148,611 |
| | Telephone | 91840* | 330 | 165 | 165 | 45 | 285 | 44 | 85,057 | 37,425 |
| Consultant psychiatrist. Consultation, more than 75 minutes | Face-to-face | 308 | 385 | 191 | 192 | 64 | 377 | 47 | 21,076 | 9,906 |
| | Video | 91831 | 380 | 191 | 189 | 69 | 309 | 40 | 5,403 | 2,161 |
| | Telephone | 91841* | 300 | 191 | 109 | 34 | 294 | 13 | 3,728 | 485 |
| Consultant psychiatrist, prepare a management plan, more than 45 minutes | Face-to-face | 291 | 556 | 406 | 144 | 66 | 354 | 37 | 29,852 | 11,045 |
| | Video | 92435 | 635 | 406 | 229 | 100 | 504 | 43 | 17,849 | 7,675 |
| | Telephone | 92475 | 526 | 406 | 120 | 60 | 234 | 16 | 3,342 | 535 |
| Consultant psychiatrist, attendance, new patient (or has not received attendance in preceding 24 months), more than 45 minutes | Face-to-face | 296 | 440 | 234 | 206 | 116 | 351 | 85 | 108,379 | 92,122 |
| | Video | 92437 | 450 | 234 | 216 | 150 | 376 | 79 | 29,632 | 23,409 |
| | Telephone | 92477* | 430 | 234 | 196 | 94 | 276 | 53 | 3,522 | 1,867 |

*Discontinued MBS items

**Comparison of the out-of-pocket costs of Medicare-funded telepsychiatry and face-to-face consultations: a descriptive study**

*Supplementary Table 2 Proportion of OOP payment according to consultation modes.*

| Service | Service count (N) | Count of services with OOP payment | Proportion of OOP payment (%) |
|---|---|---|---|
| Face-to-face | 1,039,156 | 759,386 | 73% |
| Video | 434,604 | 322,375 | 74% |
| Telephone | 432,882 | 193777 | 45% |
